# COPING STRATEGIES USED BY LATE-MIDLIFE AND OLDER ADULTS DURING THE COVID-19 PANDEMIC

**DOI:** 10.1093/geroni/igae098.2328

**Published:** 2024-12-31

**Authors:** Maria Kurth, Heidi Igarashi, Carolyn Aldwin

**Affiliations:** The Pennsylvania State University, University Park, Pennsylvania, United States; Oregon State University, Corvallis, Oregon, United States; Oregon State University, Corvallis, Oregon, United States

## Abstract

Despite greater physiological vulnerability during the COVID-19 pandemic, older adults’ self-reported mental health better than that of younger adults, perhaps due to use of more adaptive coping strategies. Compared to younger adults, older adults more frequently used strategies such as positive reappraisal (Willey et al., 2022) and fewer maladaptive ones, such as substance use (Park et al., 2020.) However, few studies have examined age differences in coping across later life. We used open-ended responses from an online survey, collected April 28 – May 5, 2020, about how adults 50+ in Oregon (N = 235, Mage = 71.27) coped with a COVID-19 difficulty in the past week. An existing scheme (Aldwin et al., 1996) guided a directed content analysis and age was categorized into three groups: late-midlife (50-64), young-old (65-74), and old-old (75). Nearly all participants (93%) responded to the coping question. The final scheme had eight codes (Krippendorf ∝=.89). The most prevalent strategies were problem-focused (49%), social support (31%), and cognitive reappraisal (27%). Three new strategies were identified: activity substitution (8%), emotion processing (4%), and proactive coping (1%). Emotion processing was more frequently by those in late-midlife (16%), compared to both young-old and old-old participants (2%), p <.01 and.05, respectively. While problem-focused coping was positively correlated with trait resilience, activity substitution was positively related to coping efficacy. Thus, even throughout later life, older adults’ use of a variety of adaptive coping strategies to manage problems early on during the COVID-19 pandemic likely contributed to their psychological resilience.

